# Lumbar Extradural Angiolipoma: Clinical Presentation and Management

**DOI:** 10.7759/cureus.12380

**Published:** 2020-12-30

**Authors:** Tochukwu Ikpeze, Abigail Kulp, Devin Williams, Aaron Huber, Addisu Mesfin

**Affiliations:** 1 Emergency Medicine, Hospital Corportation of America Kingwood, University of Houston, Houston, USA; 2 Orthopaedics and Rehabilitation, University of Rochester Medical Center, Rochester, USA; 3 Pathology, University of Rochester Medical Center, Rochester, USA

**Keywords:** angiolipoma, lumbar spine, primary spine tumor, extradural, benign

## Abstract

Angiolipomas are rare primary benign tumors that can arise in the epidural canal and cause stenosis. Of the few cases of spinal angiolipomas described, most lesions have been located in the thoracic spine, and presentation of angiolipoma in the lumbar spine is very rare. The surgical management of a 39-year-old morbidly obese woman with angiolipoma that caused stenosis with neurogenic claudication and urinary changes is described. The lesion spanned L1-L2 and surgical management consisted of T12-L2 laminectomy and en-bloc resection of the lesion. During the latest follow-up, four years after the surgery, the patient’s neurological symptoms showed improvement and there was no recurrence.

## Introduction

Extradural spine tumors usually arise from metastatic spread or hematologic (lymphoma, multiple myeloma) malignancies [1]. Primary extradural spine tumors are usually associated with osseous malignancy tumors such as chordomas and osteosarcoma and benign aggressive tumors such as giant cell tumors or aneurysmal bone cysts [2,3]. Isolated extradural primary tumors arising in the epidural canal are rare. Spinal angiolipomas are very rare tumors and less than 200 cases have been described in the literature [4].

The patient was informed that data and images concerning the case would be submitted for publication and provided consent.

## Case presentation

A 39-year-old woman presented to our spine center with a two-year history of back pain and bilateral leg pain. Her pain began spontaneously. She reported pain in her upper back (left greater than right) and bilateral lower extremities (right greater than left). The pain radiated anteriorly on her left leg and posteriorly on her right. The pain improved with squatting and leaning forward, but worsened with prolonged standing or walking. She had urinary urgency and occasional urinary incontinence. She did not have bowel incontinence, no recent weight loss, and no pain that wakes her up from sleep at night. 

**Figure 1:**
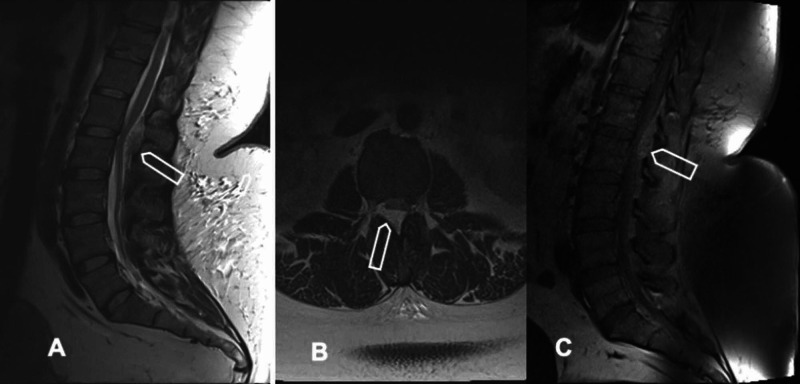
Imaging Findings (A) Sagittal T2 weighted MRI demonstrating the epidural lesion dorsal to the thecal sac spanning L1-L2 (arrow), (B) Axial T2 weighted MRI demonstrating the severe compression of the thecal sac and nerve roots (arrow), (C) Sagittal T2 weighted MRI demonstrating the hyperintense lesion (arrow),

On physical examination, the patient was afebrile, alert, and oriented. The patient had a Body mass index (BMI) of 49.8. Examination of bilateral lower extremities demonstrated normal strength except 4/5 motor strength in the right tibialis anterior. Sensation was diminished on the right leg compared to the left in the L2-S1 distribution. Radiography of the thoracolumbar spine and magnetic resonance imaging (MRI) of the lumbar spine were performed. An extradural lesion, dorsal to the thecal sac, spanning L1 - L2 and causing stenosis was noted (Figure 1). 

**Figure 2:**
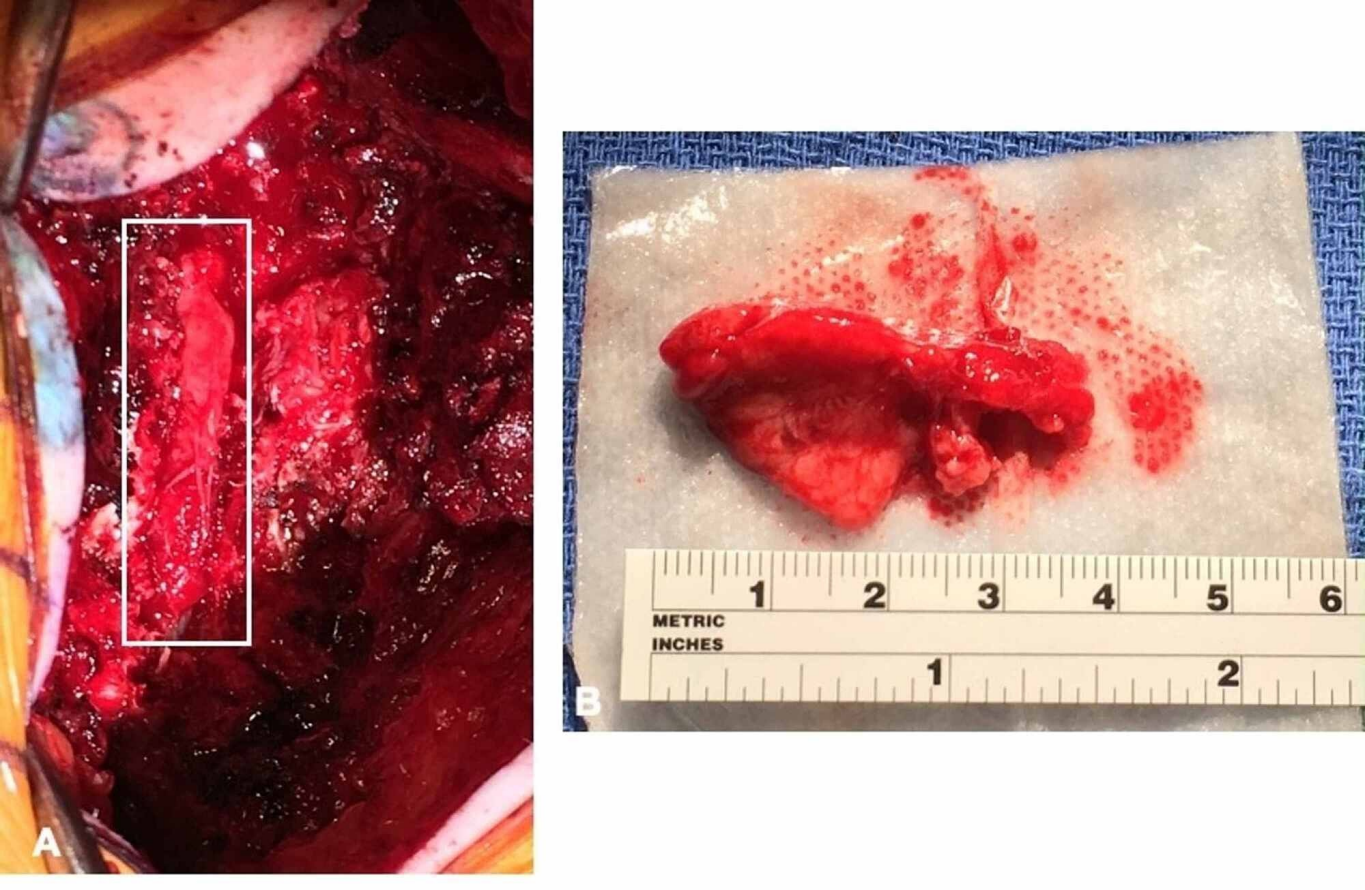
Intra-operative findings (A) Intraoperative image demonstrating the lesion (box) compression on the thecal sac. (B) The lesion that was resected en-bloc

The patient underwent T12-L2 laminectomy and en-bloc resection of the extradural lesion (Figure 2). She had an uneventful postoperative course and was discharged on postoperative day 2.

**Figure 3:**
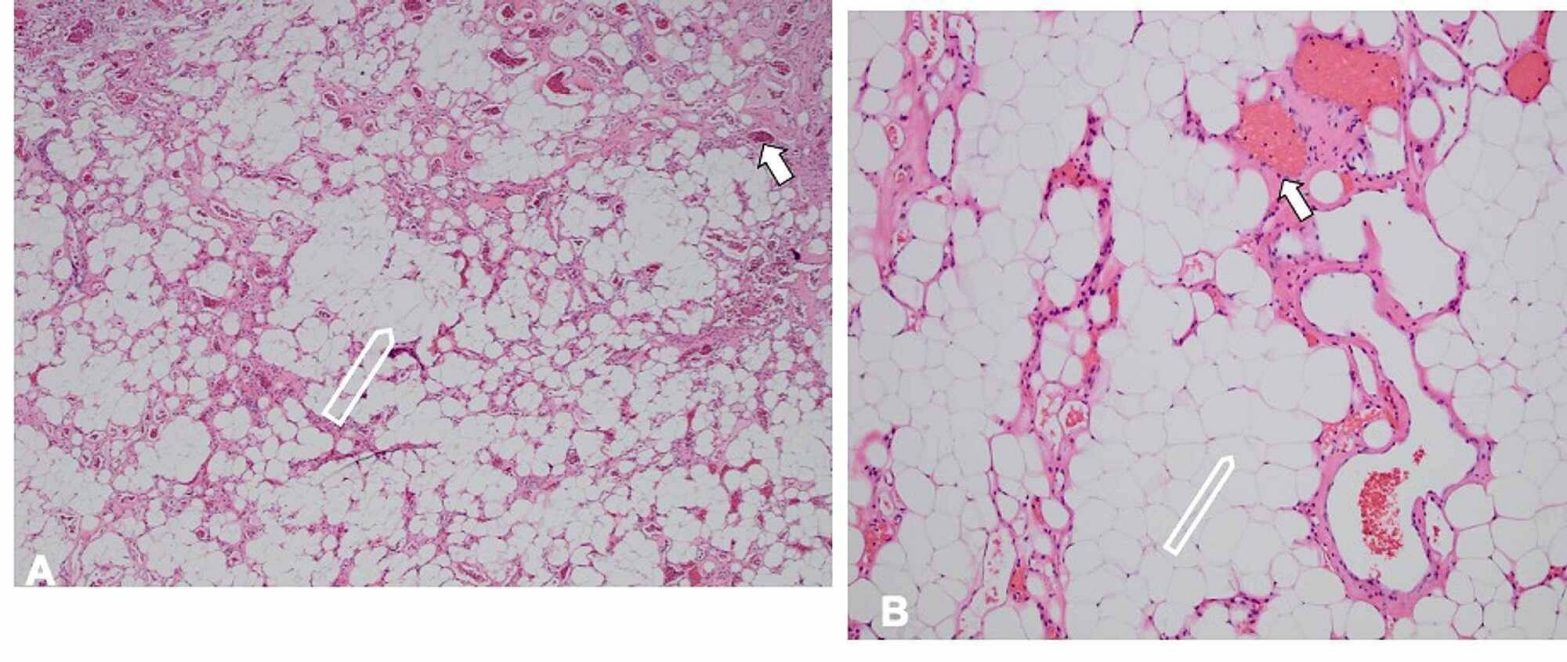
Histology findings (A) 4 x magnification and (B) 10 x magnification, hematoxylin and eosin-stained sections of the extradural lesion demonstrate a mixture of mature adipose tissue (open arrow) and variably sized, medium to small-caliber blood vessels (solid arrow). The adipocytes did not demonstrate cytological atypia. The vessels did not demonstrate cytological atypia and no mitotic figures were seen.

The patient's intraoperative biopsy indicated an angiolipoma. Hematoxylin and eosin-stained sections of the extradural tumor demonstrated a mixture of mature adipose tissue and variably sized, medium to small-caliber blood vessels (Figures 3). The adipocytes did not demonstrate cytological atypia. The vessels did not demonstrate cytological atypia and no mitotic figures were seen. 

During the latest follow-up, 48 months after the surgery, the patient showed an improvement in her urinary urgency and thoracolumbar pain with no recurrence.

## Discussion

Angiolipoma is a tumor composed of mature adipocytes admixed with an angiomatous proliferation [5,6]. Cytological atypia, nuclear pleomorphism, and mitoses in both the adipocytic and vascular components are not seen [5]. Angiolipomas are considered a variant of lipoma [6]. They may be composed nearly entirely of vascular and stromal elements with only a small lipomatous component. Alternatively, they may be composed primarily of mature adipose tissue with sparse vessels [7]. When the adipocytic component is the predominant component, capillaries with a complex branching pattern and abnormal pericytic proliferation distinguish this tumor from lipoma [7]. Additionally, spinal lipomas tend to occur in childhood, are usually located in the epidural and intradural spaces, and many are associated with congenital myelovertebral malformations [8]. When the vascular component predominates, the main differential diagnostic consideration is a hemangioma, which typically occurs in the vertebral bodies but in rare cases may occur in, or extend into, the extradural spaces [9]. Non-osseous extramedullary hemangiomas are entirely composed of multiple thin-walled or closely packed vascular channels lined by a single layer of endothelium. Hemangiomas may be of the cavernous, capillary, or venous type. Angiolipomas, in contrast, usually have some identifiable fat component [9].

Liposarcoma may be considered in the list of differential diagnoses, although these tumors rarely occur in the intradural or extradural space. However, cases of pleomorphic liposarcoma have been reported in the extradural space and demonstrate markedly pleomorphic cells including pleomorphic lipoblasts with areas of high-grade sarcoma compared to the bland and mature adipocytes of angiolipoma [10]. Myxoid liposarcoma has also been reported in the intradural and extradural spaces. These tumors usually have a myxoid background, a distinctive vascular pattern (“chicken wire” or “chicken feet”), and some signet ring-like (mononuclear) lipoblasts. This tumor should not be easily confused histologically with angiolipoma [11].

Spinal angiolipoma was first described by Berenbruch in 1890 in the case of a 16-year-old boy with numerous cutaneous lipomas when an autopsy was performed after unsuccessful surgical treatment. Spinal angiolipomas are rare, benign tumors of mature fat cells that account for 0.14% to 1.2% of spinal tumors. They cause spinal cord compression, most commonly in the epidural mid-thoracic area, and present with slowly progressing signs and symptoms of cord compression [12]. Some have termed angiolipomas that involve bone as infiltrating and angiolipomas contained within the extradural space as non-infiltrating. On MRI, angiolipomas are iso- or hyperintense on T1 and hyperintense on T2 as well as increase uptake on MRI with contrast. On the fat-suppressed sequence or STIR MRI (short tau inversion recovery MRI), some vascularity can still be seen within the tumor.

Reports on spinal angiolipomas are limited to case reports and a few case series. A female predominance has been noted. In a literature review of 108 lesions, most of the tumors (n=93, 86%) were in the thoracic spine, followed by 12 (11%) in the lumbar spine, two (1.85%) in the cervical spine, and one in the sacrum (0.9%). Most of the studies do not mention the patients’ BMI, however, one case report described a patient with a BMI of 34.3 who developed thoracic angiolipoma [13]. In a large single-center series of twelve patients with angiolipomas, Wang et al. noted eight of their 12 patients to be overweight, however, no BMI was provided [14]. In our case, the patient had morbid obesity, a BMI of 49.8, and larger series with granular data are needed to investigate the correlation, if any, between angiolipoma and obesity. 

Treatment options for angiolipoma include surgery with complete resection or subtotal removal. Both options are favorable for patients, but complete resection appears to be curative. Wider resection and radiotherapy have been described if complete removal is not easily achievable, however, radiation for this benign tumor is debatable.

## Conclusions

In conclusion, successful surgical resection of a very rare lumbar extradural epidural soft tissue tumor, angiolipoma, is described. As more cases are described and biopsy specimens collected a better understanding of the genetic etiologies contributing to this rare condition would be obtained. The presence of the lesion in the epidural canal and associated compression can result in neurological symptoms and urinary changes as described in this case.
